# Study of Adiponectin Level in Diabetic Adolescent Girls in Relation to Glycemic Control and Complication of Diabetes

**DOI:** 10.3889/oamjms.2015.098

**Published:** 2015-10-02

**Authors:** Soha M. Abd El Dayem, Hayam K. Nazif, Mona Abd EI-Kader, Maha El-Tawil

**Affiliations:** 1 *Pediatrics Department, National Research Centre, Cairo, Egypt*; 2 *Pediatrics Department, Institute of Postgraduate Childhood Studies Ain Shams University, Cairo, Egypt*; 3 *Clinical and Biochemical Department, National Research Centre, Cairo, Egypt*; 4 *Pediatrics Department, Police Hospital, Cairo, Egypt*

**Keywords:** Adiponectin, Glycemic Control, complication, type 1 diabetes

## Abstract

**AIM:**

To study the relation between adiponectin level with glycemic control and complication of diabetes.

**PATIENTS AND METHODS:**

The study included 40 female adolescent type 1 diabetic patients and 40 healthy volunteers of the same age and sex. Blood sample was taken for assessment of glycosylated hemoglobin, lipid profile and adiponectine. Urine sample was taken for assessment of albumin/creatinine ratio.

**RESULTS:**

Diabetic patients had a significantly higher diastolic blood pressure, triglyceride, total cholesterol, LDL and adiponectin than controls. Patients with diabetes complication had a significant lower BMI and HDL. On the other hand, they had higher disease duration, total cholesterol, HbA1, albumin/creatinine ratio and adiponectin. Patients with microalbuminuria had a lower BMI, higher disease duration, diastolic blood pressure and adiponectin. Patients with diabetic retinopathy had higher disease duration, insulin dose, HbA1, microalbuminuria and adiponectin. Adiponectin in diabetic patients had a significant negative correlation with BMI and positive correlation with systolic blood pressure and microlabuminuria.

**CONCLUSION:**

Serum adiponectin level is high in adolescent type 1 diabetic girls. It can be used as a predictor of diabetes complications rather than a sensitive biochemical marker for glycemic control.

## Introduction

Adiponectin is a member of a group of adipose secreted proteins, sometimes described as adipocytokines. Adiponectin is a plasma protein that has been discovered few years ago. It is produced exclusively and abundantly in adipose tissue and circulates at relatively high concentration [1]. Adiponectin has great metabolic effects including enhancement of insulin sensitivity, reduction of hepatic glucose production and decreasing gluconeogenesis [2]. In addition, plasma adiponectin levels were found to be negatively correlated with body mass index (BMI) and fat content suggesting that fat mass may exert a negative feedback on adiponectin production [3].

The circulating concentrations of adipocytokines are abnormal in type 1 diabetic children. The direction of change differs according to cytokine, pubertal development; in addition to insulin therapy and glycemic control [4]. The relationship between serum adiponectin concentration and pancreatic B cell function is a significant negative modulator for circulating adiponectin in diabetic patients and support the presence of adipoinsular axis [5].

We are aiming to determine the influence of adolescent girls with type 1 DM on circulating levels of adiponectin and to study the relation between adiponectin level with glycemic control and complication of diabetes.

## Patients and Methods

It is a cross-sectional observational study done after obtaining approval from the ethical committee of the National Research Centre, Cairo, Egypt. It conforms to the provisions of the Declaration of Helsinki (as revised in Edinburgh 2000). Written informed consent was obtained from all patients, their parents and controls after full discussion about the aim of the study.

The study included 40 female adolescents with type 1 diabetes mellitus attending the Pediatric Endocrinology Out-patient Clinic, National Research Center, Cairo, Egypt. Patients with type 1 diabetes were defined according to the criteria of American Diabetes Association [6]. Another group of 40 age matched healthy female adolescents with no obvious medical disorders and not receiving any medication were enrolled as a control group.

Inclusion criteria are type 1 diabetic females on insulin therapy with age between 9-18 years. Exclusion criteria are adolescent girls with other chronic diseases.

### Methods

All included cases were subjected to full medical history taking.

Assessment of anthropometric measures including weight in kilograms (kg), height in centimeters (cm) and body mass index (BMI) was measured as kg/m^2^ and plotted on age and sex matched Egyptian standard BMI percentiles [7].

Measurement of blood pressure (mmHg) was obtained after a 5-minute rest in the seated position using mercury sphygmomanometer. Blood pressure values were considered as hypertension if mean values (systole/diastol) were greater than 90^th^ percentile for age and sex. Blood pressure measurement was repeated twice for the validity of the reading [8].

Clinical examination include: chest, cardiac, abdomen and neurological examination. Neurological examination: Clinical neuropathy was diagnosed in patients with two or more of the following four measures: the presence of one or more symptoms of diabetic neuropathy (tingling, numbness, impaired or lost sensation), the absence of two or more reflexes of the ankle or knee tendons, a vibration perception threshold was abnormal for the patient’s age, and abnormal autonomic function (postural hypotension with a fall in systolic blood pressure of ~20 mmHg) [9].

Reviewing the records of routine annual fundus was examined by indirect ophthalmoscope through dilated pupils for assessment of diabetic retinopathy. Patient was defined as having diabetic retinopathy if the following diabetic retinal changes were present; micro-aneurysms, capillary leakage, micro-infarcts, macular edema, traction detachment of the macula or vitrous hemorrhage [10].

All patients and controls underwent the following tests: For cholesterol measurements, venous blood was sampled after a 12-h fast. Serum total cholesterol was determined by a commercial kit (Boehringer-Mannheim, Germany) [11]. High-density lipoprotein (HDL) cholesterol was separated from the serum by precipitation of the other lipoproteins with a heparin/manganese procedure [12]. Low-density lipoprotein (LDL) cholesterol was calculated using the Friedewald equation. The concentration of triglycerides(Tg) was measured in a TechnoCon AutoAnalyzer II (TechnoCon Instruments, Tarrytown, NY, USA).

Assessment of glycosylated hemoglobin % (HbA1 %); peripheral blood samples were collected on ethelene diamine tetra-acetic acid (EDTA) (1.2 ng/ml) for assay of HbA1 % using D-10 (BioRad, France) and reviewing two previous readings to calculate the mean value of HbA1 % in the last year prior to the study [13]. Reviewing the records of urinary albumin execretion in an early morning fasting urine sample was determined as urinary albumin-to-creatinine ratio (UACR) by immune-turbidimetric method (Cobas Integra SOO; Roche Diagnostics, Mannheim, Germany). Patients were diagnosed as having microalbuminuria or macro-albuminuria when UACR in at least 2 out of 3 consecutive urine samples, 2 months apart was 30-299 mg/g creatinine or ≥ 300 mg/g creatinine, respectively [14]. Adiponectin measurement by ELISA technique was also done [15].

### Statistical Methods

Statistical analysis was conducted using Statistical Package for Social Science (SPSS) program version 15.0 (Chicago, Illinois, USA). t -test for independent variables was done. Pearson correlation was also done.

## Results

The study included 40 type 1 diabetic patients, their mean age was 14.4 ± 2.5, mean duration of diabetes was 10.1 ± 2.8 and mean insulin dose was 1.4 ± 0.5 U/kg. Diabetic patients had a significantly higher diastolic blood pressure, triglyceride, total cholesterol, LDL and adiponectin than controls (Table 1). Patients with diabetic complication had a significant lower BMI and HDL. On the other hand, they had higher disease duration, total cholesterol, HbA1, albumin/creatinine ratio and adiponectin (Table 2).

**Table 1:** Comparison between demographic and laboratory data of patients and control group

	Patients group (n= 40)		Control group (n= 40)		Independent t-test	
	Mean	SD	Mean	SD	t	p-value
Age (yrs)	12.44	2.48	12.39	2.14	0.096	0.922
Weight(Kg)	46.85	12.35	44.04	12.77	-1.001	0.320
Height (cm)	146.78	15.66	142.41	14.16	-1.307	0.195
BMI (kg/m^2^)	21.60	3.98	21.33	3.71	-0.313	0.755
SBP (mmHg)	114.25	7.47	111.75	7.12	-1.532	0.130
DBP (mmHg)	74.25	5.94	71.75	6.36	-1.816	0.043
Triglycerides (mg/dl)	97.38	18.25	86.65	13.52	-2.987	0.004
Total cholesterol (mg/dl)	148.35	30.43	125.28	21.48	-3.918	0.0001
LDL cholesterol (mg/dl)	100.38	22.21	82.98	16.42	-3.983	0.0001
HDL cholesterol (mg/dl)	47.48	14.69	42.92	13.06	-1.464	0.147
CRP (µg/mL)	2.25	1.08	2.20	0.88	-0.227	0.821
Adiponectin (µg/mL)	14.63	2.20	8.11	1.50	-15.468	0.0001

BMI : Body mass index, SBP: systolic blood pressure, DBP: Diastolic blood pressure, LDL: low density lipoprotein. HDL: high density lipoprotein, CRP: C-reactive protein

**Table 2:** Comparison between demographic and laboratory data of our patients regarding diabetic complications

	Patients without diabetic complications N = 29		Patients with diabetic complications N =11		Independent t-test	
	Mean	SD	Mean	SD	t	P-value
Age (yrs)	14.09	2.71	14.86	2.17	-0.977	0.335
Weight(Kg)	49.91	14.10	43.11	8.80	1.779	0.041
Height (cm)	145.14	17.48	148.78	l3.32	-0.727	0.472
BMI (kg/m^2^)	23.33	3.70	19.47	3.28	3.450	0.001
SBP (mmHg)	115.00	7.40	113.33	7.67	0.697	0.490
DBP (mmHg)	75.00	5.98	73.33	5.94	0.880	0.384
Disease duration (yrs)	8.93	2.48	11.53	2.65	-3.195	0.003
Insulin dose (U/Kg/day)	1.26	0.41	1.58	0.62	-1.998	0.043
Triglycerides (mg/dl)	96.77	17.36	98.11	19.77	-0.228	0.821
Total cholesterol (mg/dl)	137.64	29.34	157.00	28.51	2.241	0.031
LDL cholesterol (mg/dl)	105.45	23.63	94.17	19.19	1.633	0.111
HDL cholesterol (mg/dl)	52.18	14.57	41.72	13.00	2.369	0.023
CRP (µg/mL)	2.09	0.87	2.47	1.33	-1.078	0.288
HbA1 (%)	9.42	1.55	12.19	2.79	-3.978	0.0001
Albumin/creatinine ratio (mg/gm creatinine)	18.68	5.81	130.72	104.71	-5.024	0.0001
Adiponectin (µg/mL)	13.49	1.91	16.02	1.70	-4.369	0.0001

BMI : Body mass index, SBP: systolic blood pressure, DBP: Diastolic blood pressure, LDL: low density lipoprotein. HDL: high density lipoprotein, CRP: C-reactive protein

Patients with microalbuminuria had a lower BMI, higher disease duration, diastolic blood pressure and adiponectin (Table 3).

**Table 3:** Comparison between demographic and laboratory data of our patients regarding microalbuminuria

	Patients with normo albuminoria		Patients with microalbuminuria		Independent t-test	

	N=29		N=11	

	Mean	SD	Mean	SD	t	p-value
Age (years)	14.41	2.56	14.50	2.38	-0.097	0.923
Weight (Kg)	49.12	13.11	40.86	7.72	1.955	0.058
Height (cm)	146.48	17.09	147.55	11.75	-0.189	0.851
BMI (kg/m^2^)	22.72	4.00	18.64	1.94	3.224	0.003
SBP (mmHg)	111.17	6.88	115.82	8.74	1.278	0.209
DBP (mmHg)	75.17	5.75	81.82	6.03	1.627	0.012
Disease duration (yrs)	9.21	2.69	12.45	1.68	-3.720	0.001
Insulin dose (U/Kg/day)	1.33	0.43	1.59	0.73	1.430	0.161
Triglycerides (mg/dl)	98.07	18.97	95.55	16.93	0.386	0.701
Total cholesterol (mg/dl)	150.03	29.38	143.91	34.12	0.563	0.576
LDL cholesterol (mg/dl)	101.55	22.32	97.27	22.70	0.539	0.593
HDL cholesterol (mg/dl)	47.79	15.03	46.64	14.44	0.220	0.827
CRP (µg/mL)	2.24	1.06	2.27	1.19	-0.081	0.936
HbA1 (%)	10.59	2.54	10.87	2.79	-0.303	0.764
Albumin/creatinine ratio (mg/gm creatinine)	19.00	6.23	201.18	67.52	-14.679	0.0001
Adiponectin (µg/mL)	13.85	2.05	16.69	0.78	-4.444	0.0001

BMI : Body mass index, SBP: systolic blood pressure, DBP: Diastolic blood pressure, LDL: low density lipoprotein. HDL: high density lipoprotein, CRP: C-reactive protein.

Patients with diabetic retinopathy had higher disease duration, insulin dose, HbA1, microalbuminuria and adiponectin (Table 4).

**Table 4:** Comparison between demographic and laboratory data of our patients regarding diabetic retinopathy

	Patients without retinopathy (n=29)		Patients with retinopathy (n=11)		Independent t-test	
	Mean	SD	Mean	SD	t	p-value
Age (years)	14.10	2.69	15.32	1.59	-1.401	0.169
Weight (Kg)	48.26	13.38	43.14	8.52	1.177	0.247
Height (cm)	146.59	16.83	147.27	12.76	-0.122	0.903
BMI (kg/m^2^)	22.20	3.94	19.99	3.79	1.600	0.118
SBP (mmHg)	113.79	8.20	115.45	5.22	-0.623	0.537
DBP (mmHg)	74.48	6.32	73.64	5.05	0.398	0.693
Disease duration (yrs)	9.62	2.69	11.36	2.98	-1.779	0.043
Insulin dose (U/Kg/day)	1.23	0.39	1.86	0.59	3.900	0.0001
Triglycerides (mg/dl)	99.28	17.89	92.36	19.10	1.072	0.291
Total cholesterol (mg/dl)	152.28	31.70	138.00	25.21	1.338	0.189
LDL cholesterol (mg/dl)	101.79	23.32	96.64	19.51	0.651	0.519
HDL cholesterol (mg/dl)	50.48	15.11	39.55	10.36	2.204	0.034
CRP (µg/mL)	1.93	0.84	3.09	1.22	-3.424	0.072
HbA1 (%)	9.57	1.99	13.57	1.43	-6.093	0.0001
Albumin/creatinine ratio (mg/gm creatinine)	49.69	77.97	120.27	100.70	-2.358	0.024
Adiponectin (µg/mL)	14.01	2.20	16.26	1.16	-3.204	0.003

BMI : Body mass index, SBP: systolic blood pressure, DBP: Diastolic blood pressure, LDL: low density lipoprotein. HDL: high density lipoprotein, CRP: C-reactive protein

Adiponectin in diabetic patients had a significant negative correlation with BMI and positive correlation with systolic blood pressure and microlabuminuria (Table 5). Six patients (15%) had symptoms and signs of diabetic neuropathy, 11 patients (27.5 %) had diabetic retinopathy by indirect ophthalmoscope and 11 patients (27.5%) had microalbuminuria.

**Table 5:** Correlation between serum adiponectin and different parameters in patients group

Variables	Adiponectin *(μg/ml)*	
	r	p-value
Age (years)	0.070	0.666
Weight (Kg)	-0.486	0.001
Height (cm)	0.072	0.658
BMI (kg/m^2^)	-0.767	0.0001
SBP (mmHg)	0.692	0.0001
DBP (mmHg)	0.100	0.538
Disease duration (yrs)	-0.l27	0.434
Insulin dose (U/Kg/day)	-0.056	0.730
Triglycerides (mg/dl)	-0.066	0.685
Total cholesterol (mg/dl)	-0.261	0.104
LDL cholesterol (mg/dl)	-0.175	0.279
HDL cholesterol (mg/dl)	-0.262	0.102
CRP (µg/mL)	-0.104	0.522
HbA1 (%)	0.224	0.165
Albumin/creatinine ratio (mg/gm creatinine)	0.611	0.0001

BMI: body mass mdex- SBP: systolic blood pressure-DBP: diastolic blood pressure-LDL: low density Iipoprotein- HDL: high density Iipoprotein-CRP: C reactive protein-

## Discussion

In the present study, diastolic blood pressure, cholesterol, triglyceride, LDL-c and adiponectin were significantly higher in diabetic patients than controls. On the other hand CRP as a marker of infection revealed no significant difference in diabetics and controls. Similar other studies have shown that, the absolute concentrations of total adiponectin and all subforms were higher in type 1 DM patients than healthy controls [4]. This increase in concentration of total adiponectin was primarily caused by a major increase of the high molecular weight (HMW) sub-form as reported by Barnes et al., [16] and Galler et al., [17]. Contrary to the present study, others reported no significant difference in serum adiponectin levels between type 1 diabetic patients and healthy controls [18]. The causes of the differences among the different studies are not clear, but they may be attributed to differences in ages at diagnosis, ethnic backgrounds and genetic factors such as different HLA values and autoantibody frequency [19].

Several studies showed that, circulating concentrations of adiponectin are higher in type 1 diabetic patients than in controls, while in type 2 diabetic patients, adiponectin levels are lower than controls [18, 20]. It has been proposed that this may be related to insulin’s down regulation on adiponectin secretion. Therefore, in type 1 diabetic patients, insulin deficiency may be associated with higher adiponectin levels [21]. Adiponectin has been demonstrated to have an impact on glucose homeostasis. In addition to its insulin-sensitizing effects, adiponectin may alter glucose metabolism through stimulation of pancreatic insulin secretion in vitro, suggesting that pancreatic beta cell function is one of a significant negative modulator for the circulating adiponectin level in diabetic patients and support the presence of adipoinsular axis [5].

In the current study, adiponectin had a significant negative correlation with body weight and body mass index (BMI). In the contrary a longitudinal study from Germany showed that children and adolescents with type 1 diabetes have BMI-dependent elevated serum adiponectin compared with healthy children [17]. Other study demonstrated that serum adiponectin was inversely correlated with skin fold thickness rather that BMI and it has been suggested that skin folds seemed more closely associated with adiponectin than BMI [21].

In the current study, there was no significant correlation between any of lipid parameters and serum adiponectin level. A study done by Patel et al., [22] showed no correlation between adiponectin level and carotid intimal medial thickness (CIMT). However, another study showed that, adiponectin is considered to be inversely related to obesity and dyslipidemia, and low levels of adiponectin have been linked to cardiovascular disease. The link between adiponectin and the metabolic syndrome (or abdominal obesity and low HDL cholesterol) is concealed by the overall increase of adiponectin due to diabetes complications [23].

In the present study, no significant correlation was found between serum adiponectin and HbA1. Similar finding was demonstrated by a recent study [19]. On the contrary, another study showed that, serum adiponectin level in patients with poor glycemic control is higher than in patients with good glycemic control and has direct association with HbA_1_ % in type 1 diabetics, so it can be used as a good marker for metabolic state among these patients [8]. Also other studies found such correlation with the HbA_1_% [4, 24]. A study done by Wang et al., [25] put an explanation for the effect of glycemic control on serum adiponectin levels. They found that the collagenous domain of the adiponectin molecule has four conserved lysines. In diabetic patients, glycosylation of these molecules with constant hyperglycemia could lead to a modified adiponectin molecule and an altered adiponectin function. Consequently, this could lead to diminished negative feedback, and thus to increased adiponectin concentrations in diabetics.

In the present study, 45 % of our diabetic patients had one or more of diabetic complications (diabetic neuropathy, retinopathy and or nephropathy). Patients with diabetic complications had a significant lower body weight, BMI and serum HDL. On the other hand, they had a significant higher insulin dose, disease duration, total cholesterol, HbA1, urinary albumin/creatinine ratio and serum adiponectin. This is in agreement with Morales et al., [18], Galler et al., [17] and Lindstrom et al., [26] who found the same results, in accordance with our results they reported that, serum adiponectin level was significantly higher in type 1 diabetic patients with longer disease duration (> 10 years) and they attributed high adiponectin level to renal function deterioration related to diabetes duration. Similar another Egyptian study evaluated adiponectin levels in children and adolescents with type 1 diabetes and relationship to long term complications. This study revealed that serum adiponectin was significantly elevated; urinary albumin/creatinine ratio and mean HbA1 were significantly higher in complicated patients. Moreover, patients with nephropathy showed higher values of adiponectin. So, elevated adiponectin level in adolescent girls with type 1 diabetes indicated development of complications, especially nephropathy [24]. Furthermore, Hadjadj et al., [27] reported that elevated adiponectin observed in diabetics with microvascular and macrovascular diseases; nephropathy, retinopathy, neuropathy and in a single patient with cardiomyopathy; that revealed an altered regulation of this adipocytokine in patients with complications associated with type 1 diabetes.

Patients with microalbuminuria had a significant longer disease duration and higher serum adiponectin level compared to normoalbuminuric patients. This in accordance with Hovind et al., [28] who mentioned that the development of microalbuminuria in patients with type 1 diabetes usually begins 5 to 15 years after the onset of diabetes and then increases over time. A small proportion of patients develop microalbuminuria within less than five years of disease onset Moreover, in a systematic review of nine longitudinal studies examining microalbuminuria in 7938 patients with Type I DM, the overall prevalence of microalbuminuria was (28 %) at a mean duration of diabetes of 15 years. In another report, the prevalence of microalbuminuria reached (52%) at 30 years [29]. Adiponectin probably increases as a compensatory response in these patients with microvascular complications and acts as a physiological response to restrict endothelial damage. However, later adiopnectin clearance decreased and the kidney may develop secondary resistance to adiponectin with further increase in its serum level [30].

In the current study, 11 patients (27.5%) had diabetic retinopathy. Patients with diabetic retinopathy had a significant higher insulin dose, disease duration, HbA1, urinary albumin/creatinine ratio and serum adiponectin. Chronic hyperglycemia is thought to be the primary cause of diabetic retinopathy, this hypothesis was supported by diabetes Control and Complications Trial (DCCT), which found that intensive insulin therapy, achieving a mean HbA1 % of 7.9 %, reduce the incidence of retinopathy by as much as 76% compared with conventional therapy. The reduction was directly related to the degree of glycemic control as estimated by HbA1 % [31].

Habeeb et al, [24] and Zieta et al [32] reported that adiponectin was elevated in patients with diabetic retinopathy. On the other hand, Yilmaz et al., [33] showed that adiponectin concentrations were lower in patients with diabetic retinopathy. On the other hand, Horta et al., [34] suggested that plasma levels of adiponectin did not differ between patients with and without diabetic retinopathy.

Nagaoka et al., [35] reported that adiponectin induced vasodilatation of the retinal arterioles through activation of adipo R1 and/or adipo R2 in the retinal endothelial cells, and adiponectin may be involved in retinal blood flow (REF) regulation. Because the RBF is impaired in early-stage in patients with diabetic retinopathy, therapeutic interventions that enhance the actions of adiponectin may lead to a novel potential treatment. It remains to be clarified whether increased concentrations of adiponectin are pathogenically related to the development of diabetic retinopathy or merely a beneficial counter-regulatory response. A recent meta-analysis on association between adiponectin concentration and diabetic retinopathy found a significant association and revealed high heterogeneity may be because of the plasma adiponectin levels are influenced by various factors such as; degree of obesity, age, blood lipid, gender, glucose level, kidney function, pharmacological therapy with thiazolidinediones [36].

Finally, serum adiponectin was found to have highly statistically significant negative correlations with weight and BMI in female adolescent with type 1 diabetes. This is in agreement with Kishida et al. [37] who found such negative correlation.

We conclude that serum adiponectin level is high in adolescent type 1 diabetic girls. It can be used as a predictor of diabetic complications rather than a sensitive biochemical marker for glycemic control.
